# Pediatric complex regional pain syndrome: a review

**DOI:** 10.1186/s12969-016-0090-8

**Published:** 2016-04-29

**Authors:** Rotem Weissmann, Yosef Uziel

**Affiliations:** Pediatric Rheumatology Unit, Department of Pediatrics, Meir Medical Center, 49 Tshernichovsky St., Kfar Saba, 44281 Israel; Sackler School of Medicine, Tel Aviv University, Tel Aviv, Israel

**Keywords:** Complex regional pain syndrome, Pediatric, CRPS, Pain amplification syndrome, Chronic pain

## Abstract

Complex regional pain syndrome (CRPS) is a chronic, intensified localized pain condition that can affect children and adolescents as well as adults, but is more common among adolescent girls. Symptoms include limb pain; allodynia; hyperalgesia; swelling and/or changes in skin color of the affected limb; dry, mottled skin; hyperhidrosis and trophic changes of the nails and hair. The exact mechanism of CRPS is unknown, although several different mechanisms have been suggested. The diagnosis is clinical, with the aid of the adult criteria for CRPS. Standard care consists of a multidisciplinary approach with the implementation of intensive physical therapy in conjunction with psychological counseling. Pharmacological treatments may aid in reducing pain in order to allow the patient to participate fully in intensive physiotherapy. The prognosis in pediatric CRPS is favorable.

## Background

Musculoskeletal pain is the most common reason for referral to pediatric rheumatologists. Causes of chronic musculoskeletal pain include a wide variety of inflammatory or non-inflammatory conditions, such as arthritis, hypermobility, fibromyalgia, growing pains, complex regional pain syndrome (CRPS) and more. Amplified musculoskeletal pain syndrome (AMP) is a generic and descriptive term used to describe chronic pain syndromes of unconfirmed etiology, such as fibromyalgia and CRPS. In AMP, pain signals are amplified, thus mildly painful or non-painful stimuli are registered by the body as very painful, and this leads to a functional disability – as the patient tries to avoid the induction of pain.

In this review we will focus on the condition of pediatric chronic peripheral pain, more commonly referred to as pediatric complex regional pain syndrome (CRPS). Various nomenclature are used to describe this condition, including complex regional pain syndrome (CRPS), reflex sympathetic dystrophy, reflex neurovascular dystrophy, causalgia, and localized idiopathic pain. Matles was the first to describe a case of reflex sympathetic dystrophy in a child [1].

CRPS is a condition of chronic peripheral pain, usually of a distal extremity. This syndrome is characterized by spontaneous or stimuli-induced pain, which is amplified to a very high visual analog scale (VAS) score, disproportionate to the actual incident trauma/stimulus, in the presence of a wide variety of autonomic and motor disturbances. CPRS is divided into two types. CRPS type I, previously known as reflex sympathetic dystrophy, usually develops after a preliminary event; usually minor a trauma or fracture, without any significant nerve injury. In contrast, CRPS type II, which is very rare in childhood, previously named causalgia, is induced by partial injury of a nerve or one of its major branches [2]. This review summarizes the current information regarding the epidemiology, etiology, diagnosis, treatment and prognosis of pediatric CRPS.

### Epidemiology

Among the pediatric population (children under 18 years of age), CRPS type I is more common among girls. The mean age at diagnosis is around 12 years of age. The lower extremity is more commonly involved than the upper, with some reports stating a predilection towards involvement of the foot [3–6].

Information regarding the epidemiology of pediatric CRPS type II is wanting. There are case reports of CRPS type II among children as young as 3 years of age [7–9].

Currently, there are no data available regarding the incidence of pediatric CRPS.

### Etiology and pathogenesis

The specific causes of CRPS are unknown. There is a general paucity of studies among the pediatric population regarding the etiology and pathogenesis of CRPS, although a variety of studies among adult patients present interesting hypotheses. However, the pathogenesis of pediatric CRPS is not necessarily identical to that of adult CRPS.

#### Trauma

In many cases, CRPS follows a relatively minor trauma, usually a sprain, twist, dislocation or soft tissue injury. In some cases, no previous injury was recalled [3–6, 10–13]. Fractures are the precipitating event in about 5–14 % of cases and surgical procedures in 10–15 % [14].

#### Psychological factors

Stress has an important role in inducing or perpetuating CRPS. Cruz et al. found that children with CRPS showed generally intact cognitive function. However, they demonstrated an elevated risk of somatic symptoms and emotional distress, especially anxiety [15]. These findings correlate with those of Logan et al., who found that children with CRPS reported greater functional disability and more somatic symptoms than children with other pain conditions [16].

Children with CRPS find school more stressful, commonly due to a tendency toward over achieving or to learning difficulties, and tended to have more school related problems [17, 18]. Absence from school is common.

Several studies have found a higher tendency towards a recent history of stressful life events among children with CRPS [5, 18, 19]. Furthermore, they sometimes have difficult family environments, where the child has an inappropriate role within the familial setting [18, 20]. Sherry and Weisman [18] evaluated 21 families with children diagnosed with CRPS. They identified two distinct types of families, the first with high levels of cohesion, expression and organization and with low to average levels of conflict, and the second with high overt conflict levels and low levels of family cohesion, expressiveness and organization. All 21 families had parental enmeshment with their child. They described several different stressors in the children’s lives, such as marital discord, significant school problems, and sexual abuse. However, testing did not identify any major psychopathology, with the exception of one child who scored high in somatization. They concluded that CRPS is frequently a stress related disease, which must be taken into account when treating these children.

#### Abnormal neurologic findings

The literature suggests several neurological mechanisms for CRPS, such as central sensitization and alterations in the central nervous system (CNS) and small fiber changes [21]. In central sensitization, after peripheral tissue damage or a nerve injury, a process occurs in the CNS in response. This process causes a lower threshold of generating pain and an increase in pain duration, amplitude and spatial distribution. Lebel et al. conducted a study of fMRI in twelve pediatric CPRS patients. The results demonstrated that the children had abnormal activation of the sensory cortex, motor regions, emotional processing centers and pain sensory regions after applying stimuli to the affected limb, and when the non-affected limb was stimulated. These abnormal patterns of CNS activation were present even after symptomatic recovery from CRPS. These results suggest that the brains of CRPS patients respond differently to normal stimuli [22]. Similarly, in a study by Linnman et al., of eight patients with CRPS, ages 9 to 18 years, fMRI (functional magnetic resonance imaging) imaging done in the symptomatic painful state and at follow-up in the recovered state demonstrated stimulus-induced increases in functional connectivity. They concluded that some changes in the brain persist, especially in the amygdala and basal ganglia, even after symptomatic recovery [23]. Likewise, another study investigated the habenula, a small brain structure located at the posterior end of the medial dorsal thalamus, in 12 pediatric CRPS patients using fMRI, and found that compared to control patients exhibited overall decreased habenula resting-state functional connectivity with the rest of the brain, particularly the forebrain areas [24]. Similar findings corresponding with central sensitization were demonstrated in studies of adult CRPS patients [25–27].

Small fiber changes have been suggested as a mechanism of CRPS [28]. Acquired small fiber polyneuropathy (SFPN) develops when damage occurs to small diameter unmyelinated to thinly myelinated peripheral nerve fibers that conduct pain signals. Oaklander and Klein performed a study of 41 pediatric patients with chronic widespread pain and found that 59 % had definite SFPN. An additional 17 % had probable SFPN and 22 % had possible SFPN [29]. These findings indicate that SFPN might be a common pathogenic process of pediatric chronic pain syndromes; however, further research is needed. Similarly, a study of 18 adult CRPS-I patients was performed with quantitative mechanical and thermal sensory testing (QST) followed by quantitation of epidermal neurite densities within PGP9.5-immunolabeled skin biopsies. QST revealed mechanical allodynia and heat-pain hyperalgesia at the site affected by CRPS. Axonal densities were reduced at the site of 17 of the patients compared to all the control subjects, who had no decreases in painful-site neurite reductions. These results support the hypothesis that CRPS-I is caused by persistent minimal distal nerve injury affecting nociceptive small-fibers [30].

#### Inflammatory and immune abnormalities

Alexander et al. suggested that activation of the immune system plays a role in CRPS pathophysiology. They compared plasma levels of cytokines, chemokines, and their soluble receptors among 148 CRPS adult patients to 60 age-matched, healthy controls. The subjects with CRPS were found to have significant increases in plasma cytokines and chemokines levels, and their soluble receptors as compared with controls [31]. Similarly, another study showed increases in pro-inflammatory cytokines interleukin-1b (IL-1b), interleukin-6 (IL-6), but not in tumor necrosis factor alpha (TNF-a) in the cerebrospinal fluid (CSF) of 24 CRPS patients ages 17–60 years, compared 16 controls, [32].

A study performed in the Netherlands among 52 adult CRPS patients found a significantly higher seroprevalence of Parvovirus B19, especially with CRPS of the lower extremity. However, no direct connection was found between CRPS and a previous infection, with Parvovirus B19 or any of the other microorganisms screened in the study [33]. There have been several case reports of pediatric CRPS that occurred following vaccinations [34–36].

Li et al. conducted a study among 115 adult orthopedic patients with CRPS-I who underwent successful sympathetic nerve blockade for pain relief. They found a statistically significant positive history of allergies among CRPS I patients (nearly 70 %), compared to the control group (34 %). They concluded that a positive history of allergy/hypersensitivity reactions might be a predisposing condition for CRPS [37]. Currently there is no information regarding pediatric CRPS and a positive history of allergies.

#### Genetics

There have been reports of familial occurrence of CRPS, though no specific inheritance pattern or gene has been discovered [38–40]. Several studies, most of which were small in scope, have suggested an association between CRPS and human leukocyte antigen (HLA) alleles, such as HLA-B62, HLA-DQ8, HLA-DQ1, HLA-DR13 and HLA-DR2 [41–45].

Higashimoto et al. described eight children from seven families that fulfilled the criteria for CRPS-I, and who suffered from functional/dysautonomic conditions. All the children met the established criteria for mitochondrial disease, with six of the seven families meeting the criteria for maternal inheritance. They concluded that mitochondrial DNA sequence variants might predispose children towards the development of CRPS-I and other dysautonomias [46].

### Clinical findings

Pediatric CRPS patients display a variety of sensory and motor findings, which can be linked to altered response and lower threshold of the central, autonomic and peripheral nervous systems. Common clinical features include pain and higher sensitivity to painful and non-painful stimuli of the affected limb, autonomic findings, motor disturbances, and trophic changes (changes resulting from interruption of nerve supply) [3, 5, 14, 47, 48].

#### Pain – amplification of the pain

All patients describe constant pain in the affected limb, even at rest. The pain increases with movement of the limb [5, 14, 47]. If the patient recalls a precipitating event, the pain is usually disproportionate to it [3]. Most patients suffer from allodynia (when an ordinarily nonpainful stimulus elicits pain), other common manifestations are hyperalgesia (when a mildly painful stimulus elicits intense pain) [4, 6, 14, 47]. Most patients describe their pain as burning, shooting, stabbing or electrical [14]. Low et al. described decreased range of motion (ROM) in the affected limb of all 20 pediatric CRPS patients who participated in their study [3], while Sethna et al. stated that 30 of 42 pediatric CRPS patients who participated in their study were unable or unwilling to bear weight on their affected limb due to pain [47]. Patients can present with muscle weakness and muscle atrophy varying from mild to moderate to severe, mostly due to lack of use from chronic pain [14, 47]. Patients may present with crutches or in a wheelchair due to chronic limb disuse. However, it is important to first rule out other causes for muscle weakness and atrophy, such as neuromuscular disorders, when a patient demonstrates with muscle weakness and atrophy. In such cases, one must consider a more extensive evaluation, including muscle MRI, nerve conduction tests and muscle biopsy.

#### Autonomic findings

Autonomic findings of the affected limb include swelling and edema, temperature changes (usually the affected limb is cooler), hyperhidrosis, changes in skin color, cyanosis and sensitivity to cold, and mottled, dry skin [3, 5, 47].

#### Trophic changes

With time, trophic skin changes ensue in the affected limb, such as a decrease or increase in hair and nail growth [14, 47].

#### Motor disturbances

Different motor disturbances have been described among pediatric CRPS patients, such as weakness, dystonia, tremors, spasms and fasciculations [14, 48]. Agrawal et al. described 10 CRPS patients with movement disorders that in most cases began within the first year of diagnosis of CRPS. They found that the most common motor disturbance was dystonia, predominantly characterized by tonic flexion posture, followed by tremor and myoclonus [48].

### Diagnosis

The clinical diagnosis of CRPS is based on a thorough history and physical examination, with a meticulous neurologic assessment. It is important to rule out other possible causes for chronic pain, such as orthopedic, neurological and rheumatologic disorders (Table 1). The history must include an assessment of the child’s familial, social and academic environment, such information might aid the patient’s treatment. The physical examination is usually non-revealing, with a normal neurologic examination, allodynia and signs of autonomic dysfunction might be present [20]. There are no findings in the physical examination that suggest an underlying disease as the cause of the patient’s complaints. When there is doubt in the diagnosis, the initial inquiry usually includes laboratory examination, imaging (including plain radiographs, MRI, computed tomography [CT] and bone scans) and may include an electromyography (EMG) of the affected limb.

**Table 1:** Differential diagnosis of pediatric chronic muscular pain

Diagnosis	Distinguishing charateristics
Fibromyalgia	Diffuse chronic musculoskeletal pain with multiple predictable tender points
Hypermobilty	Common, younger age (preschool to elmentary school age), pain more sever towards the end of the day, usually associated with specific activities, evidence of hypermobility in physcal examination
Myofascial pain	Pain arises from sustained contraction of a muscle, especially in the head, jaw, and upper back. Presence of a trigger point (tender point) and reproduction of the pain by maneuvers which place stress upon proximal structures or nerve roots.
Unrecognized local pathology (fracture, strain, sprain)	Trauma/strain to the affected limb, pain worsens with physical activity and excersice, positive findings in plain radiographs.
Arthritis	Inflamation of one or more joints, pain is constant, localized to the affected joint.positive physical findings.
Spondyloarthropathy	Lumbar spinal pain associated with arthritis, imaging or other evidence of arthritis affecting the sacroiliac joints and the lumbar vertebral column, response to nonsteroidal anti-inflammatory medications.
Leukemia	Child appears sick, presence of anorexia and lethargia, fever is common, nucturnal pain and bone pain. Abnormal blood count, relative thrombocytopenia, and elevated erythrocytes sedimentation rate.
Spinal cord tumors	Slow progression of pain, pain quality – low and steady intesity, abnormal neurologic examination, pathologic MRI.
Chronic recurrent multifocal osteomyelitis	Chronic, noninfectious inflammation in the metaphyses close to the physes of multiple bones. Bony tenderness over the affected sites. Presence of lytic lesions on plain radiographs. Lesions appear on bone scan. Pain usually responds to nonsteroidal anti-inflammatory drugs or corticosteroids.
Raynaud’s disease	Cold or emotional stress causes vasospasms which enduces discoloration of the fingers, toes, and occasionally other areas. Episodes are short lived, pain, numbness, or tingling can be experienced with the episode. Pain can be reproduced with a cold challenge. Digital tip ulcers might occur.
Farby disease	Deficiency of ceramide trihexoside α-galactosidase, X-linked recessive inheritance. Episodic excruciating burning pain in the hands and feet. Symptomes usuall begin in adolescence. Presence of bluish maculopapular hyperkeratotic lesions around the perineum, elevated erythrocytes sedimentation rate.
Erythromelalgia	Rare disorder characterized by burning pain, warmth, and redness of the extremities. Can be familial or secondary to myeloproliferative disorders. Pain alleviated by cold exposure.
Pernio	Episodic inflammatory skin condition presenting after exposure to cold as pruritic and/or painful erythematous-to-violaceous acral lesions, recures with cold exposure.
Chronic compartment syndrome	Usually occurs in athletes, repetitive loading or exertional activities cause exercise-induced pain that is relieved by rest. Onset of symptoms typically occurs at a specific exercise distance or time interval or intensity level, symptoms tend to subside with rest and are minimal during normal daily activities.
Peripheral mononeuropathy	More common among adults. Occurs following an injury or infection. Can cause severe burning pain in the distribution of the involved peripheral nerve. Findings in a physical examination are limited to the area supplied by the injured nerve.
Progressive diaphyseal dysplasia	Begins in adolescence. Causes severe leg pain, fatigue, headaches, weight loss, weakness, abnormal waddling gait. Diagnosis confirmed via plain radiographs which demonstrate cortical thickening and sclerosis of the diaphysis of the long bones.
Idiopathic juvenile osteoporosis	Uncommon, typically occurs just before the onset of puberty, pain in the lower back, hips, and feet, often accompanied by difficulty walking, fractures of the lower extremities can occur. Plain radiographs demonstrate low bone density, fractures of weight-bearing bones, and collapsed or misshapen vertebrae. Bone scans can demonstrate microfractures.
Thyroid disease	Hyperthyroidism/hypothyroidism can cause widespread musculoskeletal pain. History and physical examination reveals signs and symptomes of thyroid disease. Abnormal thyroid function tests.
Vitamin D deficiency	Uncommon in developed countries. Causes limb pain. Low levels of vitamin D in laboratory tests.

Baseline laboratory tests include a complete blood count, blood chemistry, C-reactive protein (CRP), erythrocytes sedimentation rate (ESR), creatinine kinase and antinuclear antibody (ANA). Usually CRPS patients have normal laboratory values. Imaging findings are normal, however, if the duration of the disease is prolonged or the degree of disability is very high, imaging modalities can demonstrate osteoporosis due to disuse [20]. A bone scan might report decreased isotope uptake of the affected limb. MRI can reveal marrow edema [49].

There is no gold standard diagnostic test for CRPS in the pediatric or adult population. Therefore, several diagnostic criteria have been developed. In 1994, the International Association for the Study of Pain (ISAP) proposed a new taxonomy and diagnostic criteria for CRPS type I and II (Table 2) [2]. However, the ISAP criteria were found to lack specificity and internal validity, thus leading to over-diagnosis of CRPS [50–52]. In 2003 the Budapest Criteria were formed as an attempt to develop more specific and sensitive criteria on the basis of history and clinical findings (Table 3) [53]. The Budapest Criteria were validated and found to have a sensitivity of nearly 100 % and specificity of 70–80 % in the adult population [54]. These criteria have not been formally validated in the pediatric population.

**Table 2:** IASP diagnostic criteria for CRPS [2]

CRPS I	CRPS II
1) The presence of an initiating noxious event, or a cause of immobilization. 2) Continuing pain, allodynia, or hyperalgesia with which the pain is disproportionate to any inciting event. 3) Evidence at some time of edema, changes in skin blood flow, or abnormal sudomotor activity in the region of the pain. 4) This diagnosis is excluded by the existence of conditions that would otherwise account for the degree of pain and dysfunction.	1) The presence of continuing pain, allodynia, or hyperalgesia after a nerve injury, not necessarily limited to the distribution of the injured nerve. 2) Evidence at some time of edema, changes in skin blood flow, or abnormal sudomotor activity in the region of the pain. 3) This diagnosis is excluded by the existence of conditions that would otherwise account for the degree of pain and dysfunction.
Note: Criteria 2–4 must be satisfied.	Note: All three criteria must be satisfied.

**Table 3:** Budapest clinical diagnostic criteria for CRPS [53]

All the following criteria must be met:
1) Continuing pain, which is disproportionate to any inciting event. 2) Must report at least one symptom in three of the four following categories: • Sensory: Reports of hyperesthesia and/or allodynia. • Vasomotor: Reports of temperature asymmetry and/or skin color changes and/or skin color asymmetry. • Sudomotor/Edema: Reports of edema and/or sweating changes and/or sweating asymmetry. • Motor/Trophic: Reports of decreased range of motion and/or motor dysfunction (weakness, tremor, dystonia) and/or trophic changes (hair, nail, skin). 3) Must display at least one sign at time of evaluation in two or more of the following categories: • Sensory: Evidence of hyperalgesia (to pinprick) and/or allodynia (to light touch and/or temperature sensation and/or deep somatic pressure and/or joint movement). • Vasomotor: Evidence of temperature asymmetry (>1 °C) and/or skin color changes and/or asymmetry. • Sudomotor/Edema: Evidence of edema and/or sweating changes and/or sweating asymmetry. • Motor/Trophic: Evidence of decreased range of motion and/or motor dysfunction (weakness, tremor, dystonia) and/or trophic changes (hair, nail, skin). 4) There is no other diagnosis that better explains the signs and symptoms.

As previously mentioned, the diagnosis of CRPS is a clinical one, based on a meticulous history and physical examination which includes a thorough neurologic assessment. The diagnostic criteria developed for the diagnosis of adult CRPS can further assist in the diagnosis of CRPS in children and adolescents. We recommend that all patients suspected of CRPS should also undergo baseline laboratory tests, including complete blood count, blood chemistry, CRP, ESR, creatinine kinase and ANA. When the history or physical examination arouses suspicion of an active thyroid disease, thyroid function test should be performed as well. It is also recommended to perform a plain radiograph (if not previously performed) of the affected limb to rule out any localized pathology to the bones, joints and surrounding tissue. Usually, no further diagnostic evaluations are needed if there are no abnormal findings in the initial diagnostic workup. However, if the initial diagnostic workup suggests a different diagnosis, such as infection, a rheumatic disease, or a malignancy, further laboratory and imaging test are needed.

### Treatment

The primary goals for the treatment of CRPS are pain relief and the improvement of all domains of functioning; thus, improving the patient’s quality of life. The scope of therapy includes an intensive physical therapy program combined with cognitive behavioral therapy (CBT) intervention. This often contradicts the patient’s logic, where rest is considered as means of improving his or her symptoms. A Cochrane review published in 2013 on interventions for treating pain and disability in adults with CRPS concluded that there was a lack of high quality evidence for most CRPS therapies [55]. In 2012, The American Pain Society published a position statement on assessing and managing children with chronic pain. It recommended the use of interdisciplinary treatment programs for children with chronic pain, which incorporate CBT with physical and occupational rehabilitation [56].

#### Physical therapy

Exercise and physical therapy (PT) are the main treatment cornerstones of all pediatric CRPS patients [56, 57]. However, there is no consensus regarding the duration, intensity or content of treatment. Gradually increasing aerobic activity is the gold standard therapy for CRPS. Several good outcomes with PT and other mobilization treatments for pediatric CRPS, alone and in addition to other therapeutic modalities have been reported [3, 5, 6, 58–62]. In a recent study, Dietz et al. created a simple, self-administered treatment protocol for CRPS-I pediatric patients that consisted of mobilization and massage, without additional intervention. Of 51 patients followed-up until symptom resolution or treatment failure, 89 % had a good outcome, with no or minimal residual pain [58]. Lee et al. conducted a prospective, randomized, single blind trial of PT and CBT for pediatric CRPS patients. They assigned 28 patients to receive either low frequency PT (once a week) or high frequency PT (three times a week). Additionally, both groups had weekly CBT. Treatment duration was 6 weeks. Pain and function measures improved significantly in both groups, no significant difference was found between the two groups at follow-up [59].

Very intense exercise programs have been found helpful for pediatric CRPS. For instance, Sherry et al. treated 103 children with CRPS with an intensive exercise program of up to 6 h daily, without the use of medication or nerve blocks, with a 92 % success rate. Among those patients, 49 continued follow-up for more than two years, and 88 % were still completely symptom free [6]. Brooke and Janselewitz reported complete resolution of pain in 25 of 32 children with CRPS after intensive inpatient rehabilitation, which consisted of physical, occupational, and psychological therapy, with no other medical intervention, followed by a home program [60]. A study of a day hospital pediatric pain rehabilitation program performed by Logan et al. included 56 pediatric CRPS patients who failed to improve with outpatient treatment. Treatment included daily physical, occupational and psychological therapy, for a median duration of 3 weeks. They found statistically significant improvements from admission to discharge in pain intensity, functional disability, subjective reports of limb function and occupational performance, these functional gains were maintained or further improved at follow-up [61].

#### Psychological therapy

Psychological therapies are a mainstay in the treatment of pediatric CRPS, and are recommended as an integral part of the interdisciplinary treatment approach [56]. Patients and their families should undergo psychological assessment in order to understand and properly address possible issues, whether they are individual, familial, social or academic [20]. There are no current large, prospective, blinded, placebo-controlled studies of the efficacy of cognitive and behavioral strategies for treating CRPS, in adults or children. However, the usefulness of psychological treatments has been demonstrated in many studies of pediatric patients with chronic pain [63–67]. A recent Cochrane review published in 2014 [68] discussing psychological therapies for the management of chronic and recurrent pediatric pain concluded that psychological therapies were effective in reducing pain intensity for children with headache and non-headache pain conditions; yet, long-term pain control effects were maintained only for children with headache conditions. In addition, they found limited evidence available to estimate the effects of psychological therapies on depression and anxiety in children with chronic pain.

The use of the internet as a mode of delivery of psychological interventions for children and adolescents with chronic pain and their families is an important new development. A Cochrane review published in 2015 examined the yield of remotely delivered psychological therapies in managing chronic and recurrent pediatric pain [69]. Conclusions were that remotely delivered psychological therapies, primarily via the internet, confer benefit in reducing the intensity or severity of pain in pediatric chronic pain conditions. However, the long-term effects of this treatment modality could not be ascertained due to lack of follow-up data. In addition, because only eight studies including 371 children contributed to the results there is considerable uncertainty around these estimates of effect. The final conclusion was that large scale trials are needed to fully ascertain the efficacy of remotely delivered psychological therapies. Subsequently, Palermo et al. published the first large, multicenter, randomized, controlled trial of Internet-delivered CBT for pediatric chronic pain [65]. A total of 273 adolescents ages 11–17 with mixed chronic pain conditions participated in the study along with their parents. Participants were randomly assigned to an internet-delivered CBT group (*n* = 138) or to a control group of internet-delivered education (*n* = 135). They were assessed prior to and immediately following treatment and at 6-month follow-up. Activity was increased from baseline to 6-month follow-up for the internet CBT group compared to control (*p* = 0.03). Additionally, sleep quality improved (*p* = 0.04) and parental help (*p* = 0.007) and protective behaviors (*p* = 0.001) decreased. This research opens the door to a possible widespread, easily accessible treatment modality for pediatric chronic pain patients.

#### Pharmacological treatments

To date, there are no specific pharmacological treatments recommended for pediatric CRPS and no large scale, clinical trials of any medications are being conducted.

Nonsteroidal anti-inflammatory drugs (NSAIDs) and paracetamol are common over-the-counter medications; thus, they are readily available to patients when chronic pain symptoms first appear. There are reports of the use of NSAIDS or paracetamol as an adjunct to physiotherapy, mostly in order to facilitate the child’s participation [62]. In a clinical study by Wilder et al., NSAIDS were prescribed to 50 of 70 patients; 60 % did not report decreased pain with this treatment modality [5].

Amitriptyline and phenytoin have been used in cases of pediatric CRPS, usually in addition to physical therapy and sometimes in combination with an antiepileptic drug, mostly gabapentin. Some patients reported improvement [3, 5, 12, 70].

There are few case reports describing the use of gabapentin for the treatment of pediatric CRPS patients [3, 5, 71–73]. Low et al. reported that 70 % of patients received amitriptyline, gabapentin or both, as adjuvant treatment for physiotherapy. These drugs were usually given early on, in order to facilitate physiotherapy, and were reduced and finally discontinued when symptoms subsided [3]. Small case series on the use of oral steroids in pediatric CRPS did not show any perceived clinical effect [5, 74].

Ketamine is an anesthetic with an analgesic effect; it is an NMDA receptor antagonist. It can cause psychomimetic side effects, which can produce delirium and hallucinations. There are studies among adult patients of the efficacy of ketamine infusions for CRPS [75–78]. In a study of 60 adult CRPS patients, pain scores over the 12-week treatment period were significantly lower among the group who received ketamine than the group receiving a placebo; however, at week 12, the significance in pain relief between groups was lost. In addition, the treatment did not lead to any functional improvement [78]. Moreover, additional studies show that when there is a response to ketamine treatment, the duration of its effect can be limited, from a few weeks to two months [76, 77]. One case report described an adolescent CRPS patient treated successfully with a combination of a continuous sciatic peripheral nerve block and parenteral ketamine infusion [79]. In a study of oral ketamine treatment for children with chronic pain, 5 of 12 participants reported an improvement in pain scores. These effects lasted for more than 4 weeks of ketamine treatment [80]. A few non-controlled reports support the use of bisphosphonates, mainly in early stages [81].

#### Invasive treatments

In a recent review [82] of invasive treatments for CRPS in children and adolescents, the more commonly used invasive treatments were single sympathetic blocks, followed by epidural catheters and continuous sympathetic blocks. Far less common were reports of spinal cord stimulation and pain-directed surgeries. Most invasive treatments were performed several times in an individual patient, in particular sympathetic blocks, with an average of two treatments per patient (range 1–12). Regarding the outcome of these invasive treatments, reports were of improvement in pain and functional disability scores in almost all patients. However, outcomes were seldom assessed with validated tools. Zernikow et al. concluded that there is a weak level of evidence for the use of invasive treatments for CRPS in the pediatric population [82].

To date there are no specific, large, prospective, blinded, placebo-controlled studies of invasive treatments in pediatric CRPS. There are, however, many case reports and small case studies on use of these treatment modalities. Invasive treatments for CRPS carry a risk of complications and the potential risks against the potential benefits should be considered carefully. To illustrate, a retrospective record review of 37 German pediatric CRPS patients, documented considerable polypharmacy, with each patient taking an average of 4.4 (range 1–10) medications at admission to a chronic pain inpatient program. In addition, 43 % of patients had undergone unsuccessful invasive pain treatments prior to admission, including at least 13 children who received two or more invasive treatments [83].

#### Additional treatments

Transcutaneous electrical nerve stimulation (TENS) is a noninvasive therapy method that uses low-voltage electrical current for pain relief. The use of this treatment modality has been described in the literature with regards to pediatric CRPS treatment [5, 9, 84–86]. However, the current literature has yet to provide sufficient proof of the efficacy of TENS treatment, and there are currently no prospective, blinded studies of the use of TENS.

### Prognosis

In general, pediatric CRPS patients have more favorable outcomes compared to adults. Many will have spontaneous resolution after a few months. The multidisciplinary team approach combing PT and CBT will lead to remission in most children; however, relapses are common. In a study by Sherry et al. [6], of the short- and long-term outcomes of children with CRPS I treated with exercise therapy, of 103 children 92 % improved in the 6–8 months following an intensive exercise program. 49 children continued follow-up for a mean of 5 years: 88 % of them were completely symptom free and 31 % had had a relapse of symptoms during the duration of their follow-up, which resolved with reinstitution of an exercise program. The median time to recurrence was 2 months, with almost 80 % of recurrences occurring during the first 6 months after treatment completion. In a retrospective review of 32 children with CRPS treated with intensive inpatient physical and occupation therapy in conjunction with psychological therapy, 89 % eventually had full resolution of their symptoms [60]. Relapses were reported in 7 children at follow-up, but 6 achieved full resolution of symptoms. Kachko et al. reported 14 pediatric CRPS cases, with a 93 % rate of full or partial recovery with treatment and a 29 % relapse rate [12]. In their study of 70 CRPS pediatric cases, Wilder et al. found that younger patients had a milder course than older ones, and that a younger age at the time of the injury correlated with less pain, better function, fewer remaining signs of autonomic dysfunction on follow-up and a shorter total duration of symptoms. Younger patients were also more likely to return to sports [5].

Other reports concluded that the prognosis of pediatric CRPS is not as favorable as reported [87, 88]. Tan et al. examined the quality of life in adults who have been treated for childhood-onset CRPS-I, with a median age of 13.2 years at the time of diagnosis. At follow-up the median age of the patients was 25.5 years, and 52 % of them complained of pain. Recurrence of signs and symptoms which could be attributed to CRPS-I was 63 %. An objective documentation of CRPS relapse was found in 33 % of cases. Overall, men had better outcomes than women [87].

## Conclusions

Pediatric CRPS is a chronic pain syndrome that differs from the adult version. It is more common among adolescent girls and the distal lower extremity is most commonly affected. Symptoms include limb pain, allodynia, hyperalgesia, swelling and/or changes in skin color of the affected limb, dry and mottled skin, hyperhidrosis and trophic changes of the nails and hair. The exact mechanism is unknown, although many different mechanisms have been suggested. The diagnosis is clinical, with the aid of the current adult criteria for CRPS. A complete patient history and examination are needed with judicious laboratory and radiographic tests to rule out other possible causes. Once pediatric CRPS is diagnosed, the standard care consists of a multidisciplinary approach with the implementation of intensive physical therapy in conjunction with psychological treatment. Treatment should take into consideration the child and the surrounding environment, family, and academic and social issues. Pharmacological treatments may aid in reducing pain in order to allow the patient to participate fully in intensive physiotherapy. Invasive treatments should be reserved for refractory CRPS cases, after considering the benefits versus the risks in each case. With the current knowledge and an intensive approach, the prognosis in pediatric CRPS has improved considerably. Further studies are needed in order to develop better diagnostic tools, improve understanding of this syndrome, and improve treatment outcomes and lower recurrence rates.

## Abbreviations

AMP: amplified pain syndrome
ANA: antinuclear antibody
CBT: cognitive behavioral therapy
CNS: central nervous system
CPRS: complex regional pain syndrome
CRP: C-reactive protein
CT: computed tomography
EMG: electromyography
ESR: erythrocytes sedimentation rate
fMRI: functional magnetic resonance imaging
ISAP: International Association for the Study of Pain
MRI: magnetic resonance imaging
NSAIDs: Nonsteroidal anti-inflammatory drugs
PT: physical therapy
QST: quantitative mechanical and thermal sensory testing
SFPN: small fiber polyneuropathy
TENS: Transcutaneous electrical nerve stimulation
VAS: visual analog scale
